# SpatialDDLS: an R package to deconvolute spatial transcriptomics data using neural networks

**DOI:** 10.1093/bioinformatics/btae072

**Published:** 2024-02-15

**Authors:** Diego Mañanes, Inés Rivero-García, Carlos Relaño, Miguel Torres, David Sancho, Daniel Jimenez-Carretero, Carlos Torroja, Fátima Sánchez-Cabo

**Affiliations:** Centro Nacional de Investigaciones Cardiovasculares Carlos III (CNIC), 28029 Madrid, Spain; Centro Nacional de Investigaciones Cardiovasculares Carlos III (CNIC), 28029 Madrid, Spain; Departamento de Ingeniería Biomédica, ETSI de Telecomunicaciones, Universidad Politécnica de Madrid, 28040 Madrid, Spain; Centro Nacional de Investigaciones Cardiovasculares Carlos III (CNIC), 28029 Madrid, Spain; Centro Nacional de Investigaciones Cardiovasculares Carlos III (CNIC), 28029 Madrid, Spain; Centro Nacional de Investigaciones Cardiovasculares Carlos III (CNIC), 28029 Madrid, Spain; Centro Nacional de Investigaciones Cardiovasculares Carlos III (CNIC), 28029 Madrid, Spain; Centro Nacional de Investigaciones Cardiovasculares Carlos III (CNIC), 28029 Madrid, Spain; Centro Nacional de Investigaciones Cardiovasculares Carlos III (CNIC), 28029 Madrid, Spain

## Abstract

**Summary:**

Spatial transcriptomics has changed our way to study tissue structure and cellular organization. However, there are still limitations in its resolution, and most available platforms do not reach a single cell resolution. To address this issue, we introduce SpatialDDLS, a fast neural network-based algorithm for cell type deconvolution of spatial transcriptomics data. SpatialDDLS leverages single-cell RNA sequencing data to simulate mixed transcriptional profiles with predefined cellular composition, which are subsequently used to train a fully connected neural network to uncover cell type diversity within each spot. By comparing it with two state-of-the-art spatial deconvolution methods, we demonstrate that SpatialDDLS is an accurate and fast alternative to the available state-of-the art tools.

**Availability and implementation:**

The R package SpatialDDLS is available via CRAN-The Comprehensive R Archive Network: https://CRAN.R-project.org/package=SpatialDDLS. A detailed manual of the main functionalities implemented in the package can be found at https://diegommcc.github.io/SpatialDDLS.

## 1 Introduction

Single-cell omics have represented one of the main technological advances toward the understanding of physiological and pathological states. However, the spatial context and location of cells are key elements with functional relevance that are missing with these techniques. In the last few years, spatial transcriptomics (ST) have revolutionized our ability to investigate biological processes by providing an unbiased way to understand tissue structure, cellular interaction, and function. Rather than studying cells as isolated and independent entities, it incorporates context through the spatial dimension while preserving the powerful information provided by whole transcriptome sequencing. However, due to the limitations of most available techniques, which fail to achieve single-cell resolution, computational methods are needed to identify the precise combination of cells within each spot. Deconvolution methods have been previously applied to bulk RNA-seq data in order to disentangle the cellular composition of samples from whole tissues or organs (Avila Cobos *et al.* 2018). For example, being able to quantify the different types of infiltrated lymphocytes in a given tumor starting from RNA-seq of the whole sample can serve as a very accurate method to predict the time-to-death from colorectal or breast cancer patients (Torroja and Sanchez-Cabo 2019). A natural extension of these methods is to apply them to deconvolute the transcriptomics data from each sequenced spot in ST data to estimate their exact cellular composition. There is a broad spectrum of tools which follow different approaches to solve this problem (Li *et al.* 2022), but most utilize single-cell RNA-seq (scRNA-seq) datasets from the same biological context as references, thereby addressing the issue as a supervised task. However, they usually rely on predefined markers defined either manually or through differential expression analysis, and typically have long running times that pose challenges for their practical application (Li *et al.* 2022).

**Figure 1.**
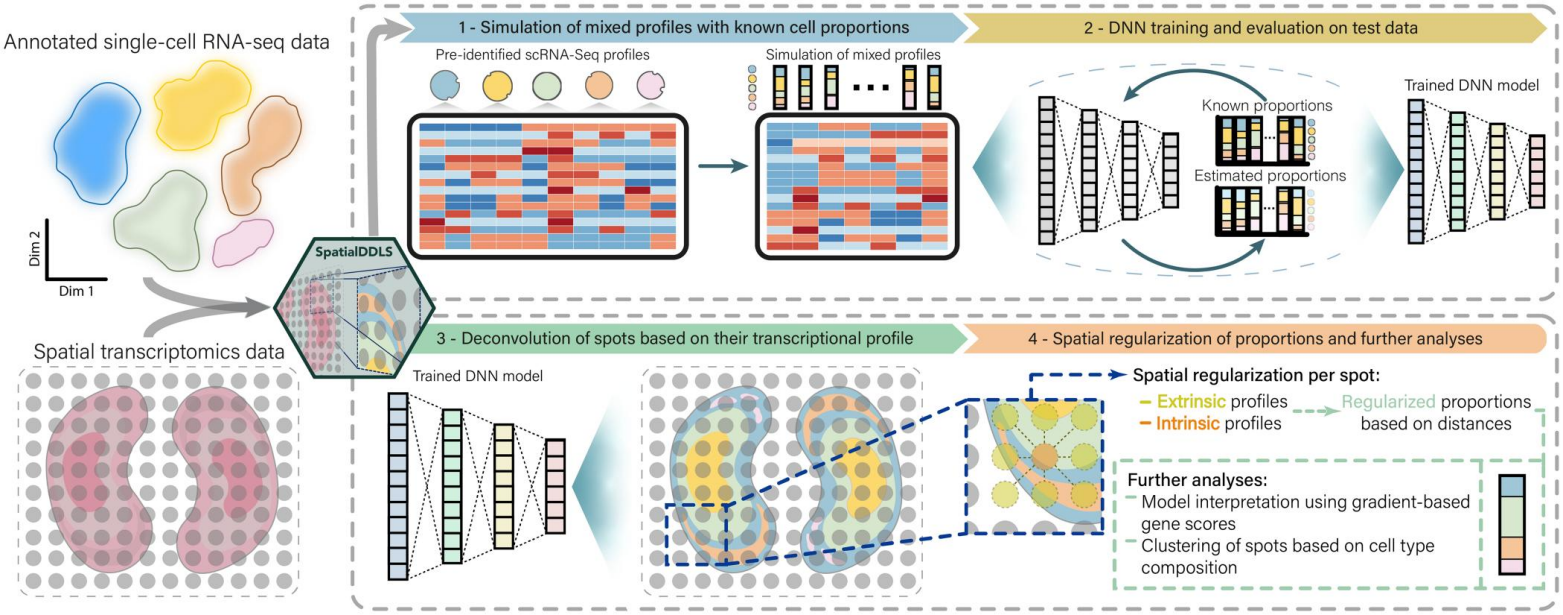
Schematic overview of SpatialDDLS. SpatialDDLS takes both an annotated single-cell RNA-seq dataset to be used as reference, and the spatial transcriptomics datasets to be deconvoluted. Then, it simulates mixed transcriptional profiles with known cell composition and trains a fully connected neural network able to make accurate predictions of cell type proportions. These predictions are adapted based on the spatial context of every spot (spatial regularization) and can be used for further analyses.

In this work, we introduce SpatialDDLS, an R package that provides a fast neural network-based solution for cell type deconvolution of spatial transcriptomics data. The algorithm employs scRNA-seq data to simulate mixed transcriptional profiles with known cell composition, with which a deep neural network (NN) is trained with the aim to uncover cell type diversity within each spot (Fig. 1). In contrast to other methods which are computationally intensive and rely on a predefined and biased set of cell type markers, SpatialDDLS does not require the definition of cell identity signatures and has a lightweight computational processing. To demonstrate its performance and efficiency, we have benchmarked our tool against two state-of-the-art spatial deconvolution methods in five ST datasets, three of which containing single-cell resolution and thus allowing a quantitative comparison. SpatialDDLS’ predictions reproduced known cell type location patterns and yielded similar results while requiring unexpensive computational resources compared to other methods, thereby making it a competitive alternative to already available tools.

## 2 SpatialDDLS

SpatialDDLS is an extension of our deconvolution tool for bulk RNA-seq (Torroja and Sanchez-Cabo 2019) implemented in the open-source R package digitalDLSorteR (Mañanes *et al.* 2022). The algorithm uses scRNA-seq to simulate mixed transcriptional profiles for training neural network models capable of estimating the cell proportions of new mixed transcriptional profiles typically present in ST data. It consists of four main steps (Fig. 1):

1. Simulation of mixed transcriptional profiles with known cellular proportions. SpatialDDLS begins by using a pre-identified scRNA-seq dataset that is partitioned into training and test cell subsets. Then, cell proportions are simulated from each labeled subset of cells, and training and test mixed transcriptional profiles are generated.
2. NN training and evaluation. A NN model is trained and evaluated using the simulated mixed profiles. Thanks to the inclusion of a test subset, this workflow allows for an assessment of whether the model is correctly identifying the transcriptional features of every cell type considered in the reference.
3. Deconvolution and spatial regularization. The trained model is then used to predict the cell composition of two sets of ST profiles:
  - Intrinsic profiles: actual transcriptional profiles of each spot to be deconvoluted.
  - Extrinsic profiles: simulated profiles generated from the k-nearest spots of every spot. This set of samples represents the transcriptional profile of the surroundings of each spot.
    The latter are used to spatially regularize the original predicted proportions by considering how similar each intrinsic profile is to its extrinsic counterpart (see Supplementary Methods, Supplementary Fig. S9a–c). This procedure assumes that cell composition/transcriptional status of every spot is influenced by its location in a tissue, and thus there is some continuity in the transcriptional profiles of neighboring spots. Consequently, this step is optional depending on the particularities of the ST dataset being analyzed, as there might be situations in which this assumption is not met.
4. Optionally, and to facilitate downstream analyses, SpatialDDLS provides a module for NN interpretation based on gradients (Simonyan *et al.* 2014), which allows to gain insights into the decision-making process of the model (Supplementary Fig. S10a–d), and a module for clustering based on predicted cell proportions (Supplementary Fig. S10e).

All these steps are implemented using the S4 object-oriented programming system of R to centralize all intermediate data generated during the workflow, making the process completely transparent and providing a user-friendly usage. Regarding its implementation, SpatialDDLS makes use of the keras (Allaire and Chollet 2021) and tensorflow (Allaire and Tang 2021) R packages for all NN-related tasks, and S4-classes from the Bioconductor’s environment (Huber *et al.* 2015) for the storage of gene expression matrices (scRNA-seq and ST). Therefore, it can be entirely integrated into the typical workflow used for analyzing transcriptomics data in R. In addition, it offers the possibility to work with The Hierarchical Data Format version 5 (HDF5) files as back-end by using the DelayedArray (Pagès 2021a) and HDF5Array (Pagès 2021b) R packages to provide a way to handle large amounts of data on RAM-constrained machines. For a detailed explanation of each step with code and examples, see the website of the package (https://diegommcc.github.io/SpatialDDLS).

## 3 Results

To evaluate its performance, we benchmarked SpatialDDLS against two state-of-the-art methods in the spatial transcriptomics field: cell2location (Kleshchevnikov *et al.* 2022) and RCTD (Cable *et al.* 2022). We chose these tools because of their superior performance in different recently published benchmarks (Li *et al.* 2022, 2023, Yan and Sun 2023). First, we analyzed two ST datasets from tissues with clear spatial cell type-distribution patterns: mouse hippocampus (Supplementary Fig. S1a) (Saunders *et al.* 2018) and mouse lymph node (Supplementary Fig. S3a) (Lopez *et al.* 2022). SpatialDDLS obtained excellent results in mixed transcriptional profiles simulated from every experiment [mean PCC = 0.97 (Pearson’s correlation coefficient) and mean CCC = 0.97 (concordance correlation coefficient) for mouse hippocampus; and mean PCC = 0.99 and mean CCC = 0.98 for mouse lymph node; Supplementary Figs S1b and S3b, respectively], indicating that the trained NN models were able to effectively detect biological signals for every cell type. Next, we compared the predictions from each method by calculating the PCC between them at the cell type level. SpatialDDLS made similar predictions to those of cell2location and RCTD, demonstrating a high PCC for the most abundant cell types of each tissue (Supplementary Figs S1c and S3c). In addition, the three tools showed similar spatial patterns of cell type proportions that indeed co-localized with the expression of their markers (Supplementary Figs S2 and S4).

Then, to perform a quantitative comparison among SpatialDDLS, cell2location and RCTD, we decided to analyze three single-cell resolution ST datasets simulating spots with a mixture of cell types by binning neighboring cells: seqFISH (Eng *et al.* 2019) (Supplementary Fig. S5a), STARmap (Wang *et al.* 2018) (Supplementary Fig. S6a), and MERFISH (Moffitt *et al.* 2018) (Supplementary Fig. S7a). The three methods yielded highly comparable predictions for the predominant cell types in each dataset (Supplementary Figs S5b, S6b, and S7b), although some differences were observed for specific cell types. For instance, SpatialDDLS outperformed cell2location and RCTD at predicting the most important cell types in the mouse neo-cortex samples (STARmap and seqFISH datasets): the excitatory L2/L3, L4, L5, and L6 neurons (Supplementary Figs S5b and c, S6b and c, and S8b). Indeed, its overall performance for these datasets was superior according to CCC and JSD evaluation metrics (Supplementary Fig. S8a), although not reflected in PCC and RMSE, the latter being better only for STARmap. Nevertheless, we believe that CCC is a more reliable metric for evaluating this problem, as it considers not only the linear relationship between two variables but also their distance to the identity (see Supplementary Methods). Altogether, the results across all datasets were comparable among the three methods, showcasing a high level of agreement. Nonetheless, specific tendencies were observed, making their predictions complementary to each other for a better understanding of the structure of the tissue under study. In contrast to other methods, SpatialDDLS offers interpretability to predicted cell proportions by reporting gradient-based gene scores that highlight important genes for the predictions (Supplementary Fig. S10). In dditiona, it incorporates functionalities that might help to understand structural features of tissues, such as visualizing distances between extrinsic and intrinsic spots to explore the spatial consistency of each region at the transcriptional level (Supplementary Fig. S9b).

Finally, we evaluated the performance of each method at the computational level (Supplementary Fig. S8c). While both SpatialDDLS and RCTD demonstrated comparable running times across all datasets, cell2location exhibited the longest durations. On the other hand, in terms of RAM consumption, cell2location was superior in performance to SpatialDDLS and RCTD. It is important to note that SpatialDDLS allocates all intermediate steps generated during the deconvolution process in order to let users explore them, although this may be dispensable if memory optimization is prioritized.

## 4 Conclusion

SpatialDDLS is a flexible spatial deconvolution tool of easy use and fully integrated in the R/Bioconductor ecosystem. We have demonstrated that it generates comparable results to those of two state-of-the-art methods while uses unexpensive computational resources that allow its implementation in the regular workflow for ST data analysis. In addition, SpatialDDLS does not need the definition of a set of markers for each cell type and performs whole-transcriptome predictions. We think that this fact can be useful in the context of paired scRNA-seq and ST datasets, as SpatialDDLS could account for specific transcriptional features that cell types may undergo depending on the biological context. Finally, even though the algorithm is based on NN models, SpatialDDLS makes the deconvolution process quite transparent thanks to two features: when a model is trained, it allows the users to check out whether every cell type is being correctly detected in simulated samples (Supplementary Figs S1b and S3b); and by making use of gradient-based interpretation techniques, SpatialDDLS integrates functions that allow to gain insights into the model’s decision-making process (Supplementary Fig. S10a–d). Overall, these features make SpatialDDLS a robust alternative to existing methods that might be useful for the field.

## Supplementary Material

btae072_Supplementary_Data

## Data Availability

No new data were generated or analysed in support of this research.
