# Supplementary material for: SpatialDDLS: an R package to deconvolute spatial transcriptomics data using neural networks
Source: Bioinformatics. 2024 Feb 15;40(2):btae072. doi: 10.1093/bioinformatics/btae072 (PMC10881086; doi:10.1093/bioinformatics/btae072)
Supplement: btae072_Supplementary_Data [file btae072_supplementary_data.pdf]

Supplementary Material: SpatialDDLS: An R package to deconvolute spatial transcriptomics data using neural networks

Diego Mañanes[1], Inés Rivero-García[1,2], Carlos Relaño[1], Miguel Torres[1], David Sancho[1], Daniel Jimenez-Carretero[1], Carlos Torroja[1], Fátima Sánchez-Cabo[1*]

[1]Centro Nacional de Investigaciones Cardiovasculares Carlos III (CNIC), 28029 Madrid, Spain.
[2]Departamento de Ingeniería Biomédica, ETSI de Telecomunicaciones, Universidad Politécnica de Madrid, 28040 Madrid, Spain.
*Corresponding author

Table of Contents

Supplementary Methods 2

SpatialDDLS overview 2

1 – Loading data 2

2 – Simulation of mixed transcriptional profiles 2

3 – Training the neural network model and deconvolution of spatial transcriptomics datsets 3

4 – Deconvolution of ST dataset and spatial regularization 3

5 – Optional 1: model interpretation 4

6 – Optional 2: cell compositional clustering 5

scRNA-seq and spatial transcriptomics datasets 5

Deconvolution of spatial transcriptomics datasets and comparison with cell2location and RCTD.... 6

Validating gradient-based gene scores 8

Code availability 8

Supplementary Figures and Tables 9

References Supplementary Material 19

Supplementary Methods

SpatialDDLS overview

SpatialDDLS is an R package which provides a user-friendly approach for deconvoluting spatial transcriptomics data. The package offers several functionalities for effectively handling large datasets through the utilization of HDF5 files and streamlines the overall process with a few simple function calls. It tries to offer a framework in which deconvolution of spatial transcriptomics data using deep neural networks (NNs) is easy and flexible depending on the particularities of each problem. The algorithm comprises four main steps.

1 – Loading data

SpatialDDLS operates within a supervised framework, requiring thus both the spatial transcriptomics (ST) data wanted to be deconvoluted and a reference single-cell RNA-seq (scRNA-seq) dataset with pre-identified cell types. The goal is to consider only those genes that are relevant in both types of data for further steps. To do so, it starts by applying two filters to reduce the original number of dimensions. These filters must be modified according to the particularities of each dataset (number of cells, sequencing depth, etc.):

1. Filtering genes at the cell level by setting a minimum count cutoff for N cells.
2. Filtering genes at the cluster level (only applied to the scRNA-seq data): it consists of a cutoff of non-zero average counts per cluster and the disposal of genes with a log fold-change (logFC) less than 0.5. Then, the top 300 genes (modifiable through the `sc.n.genes.per.cluster` parameter) with the highest logFC per cluster are kept. LogFCs are calculated per cluster with respect to the average across all cell types.

Then, mitochondrial genes are removed and only genes shared between both modalities are retained. In addition, if multiple spatial transcriptomics slides are provided, SpatialDDLS offers the option to keep only those genes present in a specified number of slides. Finally, if the number of selected genes is greater than the `top.n.genes` parameter (2,000 by default), genes are ranked based on variability across the scRNA-seq dataset and `top.n.genes` are chosen for further analyses. These procedures aim to expedite subsequent steps by avoiding the consideration of the entire noisy expression matrix. Notably, when working with massive amounts of data, single-cell profiles can be provided as HDF5 files. SpatialDDLS handles this format by using the DelayedArray (Pagès, 2021a) and HDF5Array (Pagès, 2021b) R packages.

2 – Simulation of mixed transcriptional profiles

The second step consists of the generation of mixed transcriptional profiles with known cell compositions. This is achieved by the `genMixedCellProp` function, which generates a cell composition matrix, and `simMixedProfiles`, which simulates mixed profiles and saves them in the SpatialDDLS object. SpatialDDLS is again able to accommodate these simulated samples using HDF5 files as back-end, but this option is not necessary for most situations.

In the cell composition generation process, the package initially partitions the single-cell profiles into training and test subsets (default split ratio of 0.75). The cell composition of each simulated profile is then determined by randomly sampling from a Dirichlet distribution in which a random number of cell types have been truncated to be zero. This method is designed to introduce greater sparsity in terms of cell type composition, enabling the NN model to learn from scenarios where certain cell types might be missing. For analyses similar to those presented in this article, we recommend generating a total

of 10,000-15,000 mixed transcriptional profiles (`num.sim.spots`). Each sample comprises a specific number of aggregated cells with a default of 50 single-cell profiles per sample. Finally, in cases where certain cell types are underrepresented, SpatialDDLS provides the option to simulate new single-cell profiles using the ZINB-WaVE framework (Risso et al., 2018) by the `estimateZinbwaveParams` and `simSCProfiles` functions. This feature enables to increase cell type-specific signals, thereby expanding their representation through an augmentation-based approach.

In the simulation step, single-cell profiles can be aggregated using different strategies, but aggregating raw counts by summing them up is the default one. Then, mixed transcriptional profiles are normalized to account for sequencing depth by dividing by total counts and multiplying by 10,000, and log2-transformed for variance stabilization.

3 – Training the neural network model and deconvolution of spatial transcriptomics datsets

Once the SpatialDDLS object contains normalized mixed transcriptional profiles, a NN model is trained using the training subset. To address potential issues arising from variations in gene scales, SpatialDDLS offers two transformations typically used in the NN field: rescaling between 0 and 1, and standardization, which makes features to have mean 0 and standard deviation equal to 1. Whereas the latter is the preferred option for most of the cases, for datasets where the number of dimensions is not very high (around 100 genes), we observed that rescaling may be a better choice.

The package implements a default architecture for the NN model, but users have flexibility to fully customize it based on the specific characteristics of their datasets. However, we recommend considering the following parameters as a starting point:

- Architecture: Two hidden layers with 200 neurons each.
- Activation function for hidden layers: ReLU function as activation function.
- Optimization function: Kullback-Leibler divergence, which is suitable for modeling probability distributions.
- Batch size: 64 samples.
- Number of epochs: 60-80.
- Dropout regularization: One dropout layer with a rate of 25% to every hidden layer to prevent overfitting.
- Activation function for the last layer: Softmax function, enabling the neural network to predict probabilities that can be interpreted as cell type proportions.

After training, the model becomes capable of predicting cell composition of new samples based solely on their transcriptional features. Additionally, the package also includes several functions for inspecting and evaluating the predictive performance of the model. These functions let users assess whether model are effectively learning patterns associated with specific cell types, and thus determining if adjustments to the hyperparameters are required.

4 – Deconvolution of ST dataset and spatial regularization

Once the NN is trained, SpatialDDLS uses it to deconvolute the cell composition of two sets of ST profiles inferred from the dataset to be deconvoluted:

- Intrinsic profiles: actual transcriptional profiles of every spot in the ST dataset.
- Extrinsic profiles: simulated profiles generated from the k-nearest spots of every spot.

The information contained in the second set of profiles serves to regularize the original predictions by incorporating the spatial dimension during the deconvolution process. This procedure is based on the assumption that cell composition (and thus, transcriptional status) of every spot is influenced somehow by its location in a tissue, i.e., cell composition of a spot can be predicted by its nearest spots to a certain extent. SpatialDDLS leverages this feature by simulating extrinsic profiles that represent the transcriptional profile of the surroundings of every spot. In detail, the procedure is as follows:

- First, a set of extrinsic profiles is simulated (see Section 2) from the k-nearest spots of every spot.
- A PCA-space containing both types of profiles is calculated in order to make Euclidean distances more reliable and its calculation computationally cheaper. Then, Euclidean distances between each pair of profiles are computed.
- Assuming that the spatial information is only helpful in cases where extrinsic profiles are transcriptionally close enough to their intrinsic pairs, those spots with distances further than the average are not spatially regularized.
- On the other hand, those profiles with shorter distances are regularized by calculating a weighted mean between the intrinsic and extrinsic predicted proportions. Weights are determined based on the remaining distances by rescaling them between 0 and 0.5, so that extrinsic profiles can modify the original predictions by up to a 50%.

We have observed that this spatial contextualization slightly improves the SpatialDDLS prediction performance (Figure S9a). In addition, manual inspection of the Euclidean distances and obtained predictions using the intrinsic and extrinsic profiles might be beneficial to better understand the spatial variability of each dataset (Figures S9b-c). Nonetheless, there may be situations where spatially nearby spots deviate from this assumption. For this reason, the package always returns these three sets of predictions, which can be inspected and used as a result depending on the particularities of the ST data under study (resolution of the technology used, architecture of the tissue, etc.).

5 – Optional 1: model interpretation

Despite the use of NN, which are typically considered black box models, SpatialDDLS provides a set of functions that allow users to gain insights into the decision-making process of the models. We have implemented a popular approach in the field of computer vision known as Vanilla Gradient (Simonyan et al., 2014). This algorithm consists of the calculation of the partial derivatives of the classification loss (L) or the class probabilities ($S_c(x)$) with respect to the input features (x). Let's formulate the problem for the last case:

Given a neural network, the output for a determined sample (transcriptional profile, $x \in \mathbb{R}$) can be formulated as $S_c(x)$, which is the probability of sample x of belonging to the class c (i.e., the proportion of cell type c detected in sample x). If the model was linear, the classification model could be formulated as:

$$S_c(x) = w^T x + b_c$$

Where w represents the weight vector, and b_c is the bias term. However, $S_c(x)$ is a highly non-linear function in NN models, and therefore w cannot be directly used to infer the magnitude of each feature. Instead, Vanilla Gradient tries to approximate $S_c(x)$ by applying a first-order Taylor expansion:

$$S_c(x) \approx w^T x + b$$

Where w is the derivative of this probability with respect to the input:

$$w = \frac{\delta S_C}{\delta x}|_{x_0}$$

The same reasoning can be used to calculate the gradients of the loss function of samples belonging to a specific class with respect to the inputs. However, in each case, the interpretation is different:

- If gradients with respect to the input variables are calculated using the loss function (`method = "loss"` in the `interGradientsDL` function), genes with negative gradients (those that minimize the loss function) will be positively associated with the presence of each cell type.
- Conversely, if gradients with respect to the input variables are calculated using classes (`method = "class"` in the `interGradientsDL` function), genes with positive gradients (those that make the probability of being a cell type higher) will be positively associated with each cell type.

We apply this algorithm to simulated mixed transcriptional profiles comprised only of one cell type, so that the obtained gradient-based genes scores can be directly associated with each cell type to better understand the predictions. Since each mixed transcriptional profile is made of a different set of cells from the same cell type, the resulting gradient-based gene scores encompass the biological variability inherent within a cell type. Finally, these scores are averaged at the cell type level so that a unique set of gene scores is obtained for each cell type.

These scores can be represented as heatmaps by using the `plotHeatmapGradsAgg` function (Figure S10a), and the top N genes with the highest scores per cell type can be easily obtained using the `topGradientsCellType` function. We have observed that these genes are associated with cell type markers (Figure S10b and S10d). Furthermore, the spatial representation on the ST data of their expression levels usually aligns with the presence of the respective cell types from which the scores were obtained (Figure S10c). However, it is important to highlight that due to the multi-variate nature of this approach, these genes should not be interpreted as cell type markers. Rather, gradients are surrogates at the feature level for predictions made considering all input features collectively, emphasizing how the interconnected nature of gene influences the predictions. Gradients should be interpreted with caution, although we think that they might serve to gain a better intuition and understanding of the predictions of the model.

6 – Optional 2: cell compositional clustering

Lastly, in order to facilitate downstream analyses, SpatialDDLS incorporates a function to calculate clusters based on the predicted cell proportions (the `spatialPropClustering` function) by using k-means and graph-based clustering algorithms. These clusters correspond to tissue regions with similar cell composition that might be relevant for downstream analyses (Figure S10e).

scRNA-seq and spatial transcriptomics datasets

We have analyzed a total of five paired ST and scRNA-seq datasets for this manuscript:

- <u>Mouse hippocampus</u>. scRNA-seq dataset used as reference is sourced from Saunders et al. 2018 and was downloaded from the Broad Institute's Single Cell Portal (SCP948 study). The spatial transcriptomics dataset was obtained from the 10x Genomics website (10x-Genomics, 2020).

- Mouse draining lymph node (dLN). scRNA-seq and spatial transcriptomics data were obtained from Lopez et al. 2022 and are available at the Gene Expression Omnibus database under the accession number GSE173778.
- seqFISH of mouse brain cortex. This dataset was generated by Eng et al., 2019 and was used in the Li et al., 2022 and Li et al., 2023 benchmarks. It contains measurements of 10,000 genes for 523 individual cells. The individual cells were aggregated into 72 spots containing between 1 and 17 cells each. The processed spatial data was obtained from Li et al., 2022 (Dataset 4) and is available at https://drive.google.com/drive/folders/1pHmE9cg_tMcouV1LFJFtbyBJNp7oQo9J. A Smart-seq data set from the mouse primary visual cortex (VISp) containing 14,249 cells and 34,041 genes and obtained from https://portal.brain-map.org/atlases-and-data/rnaseq/mouse-v1-and-alm-smart-seq was used as a reference. This data set contains 8 cell types.
- STAR-map of mouse visual cortex. This dataset was generated by Wang et al., 2018 and was used in the Li et al., 2022 benchmark. It contains measurements of 1,020 genes for 1,549 individual cells. The individual cells were aggregated into 189 spots containing between 1 and 17 cells each. The processed spatial data was obtained from Li et al., 2022 (Dataset 10) and is available at https://drive.google.com/drive/folders/1pHmE9cg_tMcouV1LFJFtbyBJNp7oQo9J. A Smart-seq data set from the mouse primary visual cortex (VISp) containing 14,249 cells and 34,041 genes and obtained from https://portal.brain-map.org/atlases-and-data/rnaseq/mouse-v1-and-alm-smart-seq was used as a reference. This data set contains 13 cell types.
- MERIFSH of mouse medial pre-optical area (MPOA). This dataset was generated by Moffitt et al., 2018 and was used in the Yan and Sun, 2023 benchmark. It contains measurements of 135 genes for 1,000 – 6,000 individual cells per slide. The gene expression matrix and metadata of individual cells is available at https://datadryad.org/stash/dataset/doi:10.5061/dryad.8t8s248/. 59,651 individual cells from a single slide (Animal 2, Bregma coordinates -0.09) were aggregated into 256 spots containing between 1 and 40 cells each, as described in STdeconvolve (Miller et al., 2022) and https://github.com/SunXQlab/ST-deconvoulution/blob/main/synthetic_st_dataset/synthetic_MERFISH_dataset. 52,989 single cells from an independent biological replicate (Animal 1, all slides) were used as a reference. This data set contains 9 cell types.

For basic analyses such as non-linear dimensionality reduction, Seurat v4.1 (Hao et al., 2021) was used. In brief, most variable genes were taken and used as input for principal component analysis (PCA). Then, first 20 principal components were used for visualization through Uniform Manifold Approximation and Projection for Dimension Reduction (UMAP) using default parameters implemented in Seurat.

Deconvolution of spatial transcriptomics datasets and comparison with cell2location and RCTD

Regarding SpatialDDLS deconvolution analyses, except for the mouse dLN dataset in which the `sc.n.genes.per.cluster` parameter was set to 150, and the MERFISH of mouse MPOA dataset in which data were rescaled between 0 and 1, the parameters used are those the package implements by default. For the analyses conducted using cell2location and RCTD, the default parameters and tutorial recommendations were used. Particularly:

- cell2location: We followed the tutorials available on the cell2location documentation website: https://cell2location.readthedocs.io. The single-cell regression model was trained with parameters `max_epochs=250`, `lr=0.002`. The cell2location model was obtained with parameters `max_epochs=30,000`. Then, cell2location's predictions in each spot were divided by the maximum to treat them as cellular proportions.
- RCTD: We followed the tutorials present in the GitHub repository (https://github.com/dmcable/spacexr.git). The `"full"` mode was selected.

To compare the three methods, each dataset was differently analyzed depending on the availability of ground truth. For those datasets with no ground truth, estimated cell proportions of each tool were analyzed by calculating the Pearson's correlation coefficient (PCC) of estimated cell proportions per method and cell type (see PCC definition below). Then, PCCs were clustered and plotted as a heatmap using the ComplexHeatmap R package (Gu et al., 2016). In addition, to determine if the predicted cell type proportions are consistent with the expression levels of established cell type markers, we computed the mean Z-score expression values of a manually selected set of markers for each cell type (Table S1). This analysis was performed primarily to visualize the spatial distribution of specific cell types and not for quantitative comparison purposes.

For the quantitative comparison among the three methods in datasets with single-cell resolution, the following metrics were calculated at the cell type and method levels:

- **Pearson's correlation coefficient (PCC)** for each cell type and method was calculated as follows:

$$PCC = \frac{E[(P_{ik} - \bar{P_k})(T_{ik} - \bar{T_k})]}{\sigma(P_k)\sigma(T_k)}$$

  It allows to determine whether there is a linear relationship between two variables. However, it does not take into account if these vectors are different because it is a scale invariant metric.

- **Concordance correlation coefficient (CCC)** for each cell type and method was calculated as follows:

$$CCC = \frac{2cov(P_k, T_k)}{\sigma(P_k)^2 + \sigma(T_k)^2 + (\bar{P_k} - \bar{T_k})^2}$$

  This metric is particularly useful to measure error in deconvolution tasks because of its ability to quantify the linear relationship and distance between two variables with respect to the identity, which would be a perfect performance (it is scale sensitive). Therefore, it measures the level of agreement between two variables, which is the actual objective when evaluating the model performance in this task.

- **Root-mean-square error (RMSE)** for each cell type and method was calculated as follows:

$$RMSE = \sqrt{\frac{1}{N}\sum_{i=1}^{N}(P_{ik} - T_{ik})^2}$$

- **Jensen-Shannon Divergence (JSD)** for each cell type and method was calculated as follows:

$$JSD = \frac{1}{2}\left(D_{KL}(P_k \parallel R) + D_{KL}(T_k \parallel R)\right)$$

where DKL represents the Kullback-Leibler divergence and R is $R = \frac{1}{2}(P_k + T_k)$.

In all equations, we assume that there are N spots containing up to K cell types in which the expression of j genes has been measured. T_{ik} is the real proportion of cell type k in spot i, and P_{ij} is the predicted proportion of cell type k in spot i. Regarding their interpretation, PCC and CCC are correlation metrics, and thus the greater the better. On the other hand, RMSE and JSD are distance metrics, and therefore the lower the better.

All deconvolution analyses (including SpatialDDLS) were performed with an Intel(R) Core(TM) i5-10500U CPU @ 3.80 GHz with 32 GB of RAM.

Validating gradient-based gene scores

To assess the specificity of genes with the highest gradient-based gene scores for each cell type, we examined the level of overlap between them and gene markers identified through classical univariate statistics. Particularly, we first obtained gene markers for each cell type with respect to the rest by using Wilcox test as implemented in Seurat with parameters by default and selected the top 150 significant genes (adjusted p-value ≤ 0.05) with the highest logFC. Then, we identified the top 50 genes with the highest gradient-based gene scores for each cell type. Finally, we conducted an overrepresentation analysis using the clusterProfiler R package (Yu et al., 2012) to determine whether the genes selected via the gradient-descend approach were enriched in those gene sets obtained using univariate statistics. Then, the obtained -log10(q-values) were represented as a heatmap (Figure S10b).

Additionally, we explored the number of shared genes among the top 50 genes with the highest gradient-based gene scores for each cell type by calculating the intersection sets and visualizing them as an upset plot (Figure S10d) by using the ComplexHeatmap R package. The intersection sets with a degree less than 3 were pruned from the upset plot for visualization purposes.

Code availability

The source code for SpatialDDLS is available at https://github.com/diegommcc/SpatialDDLS, and it is also available on CRAN https://CRAN.R-project.org/package=SpatialDDLS.

Supplementary Figures and Tables

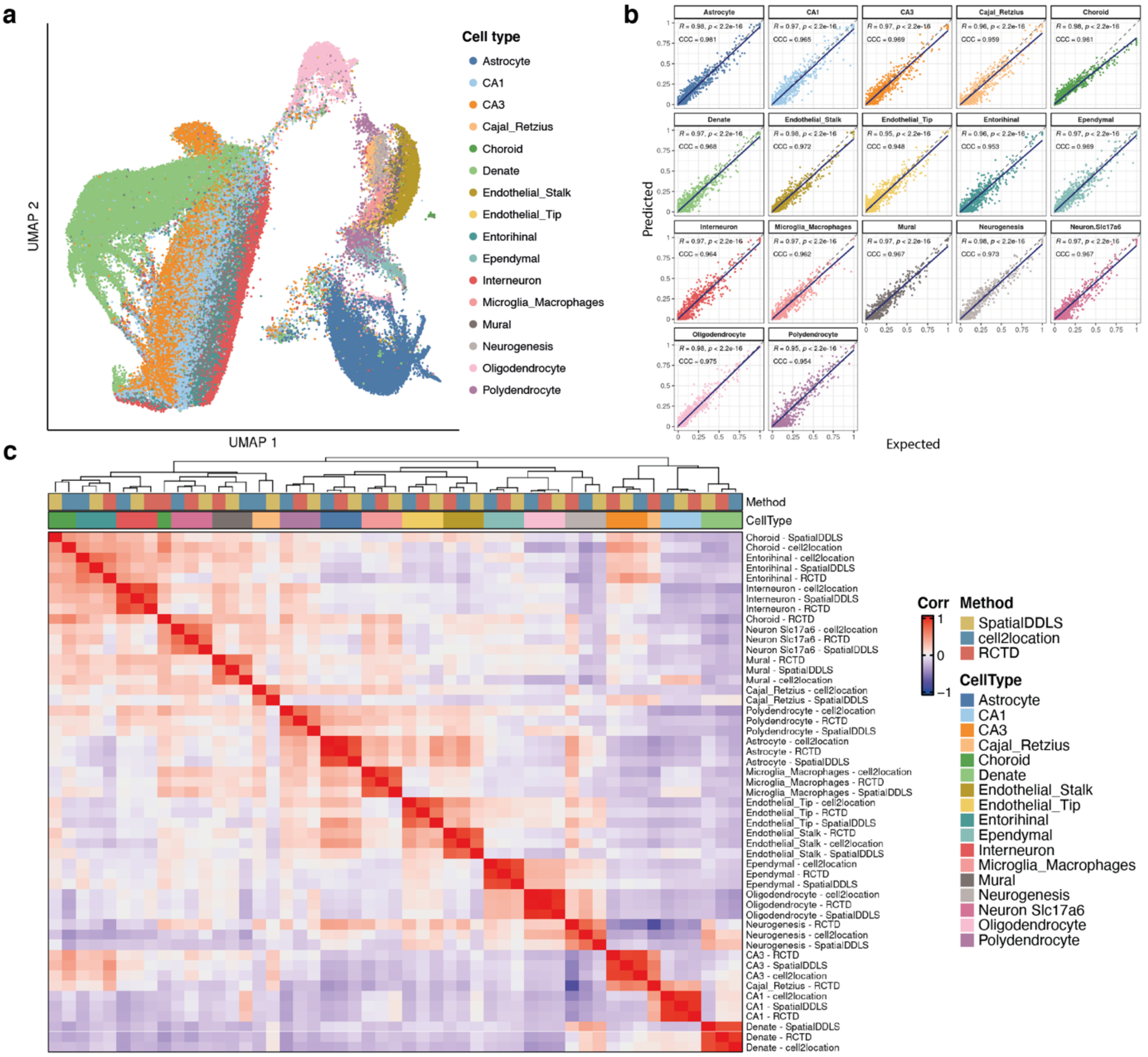

Figure S1: Mouse hippocampus dataset. a. UMAP representation of scRNA-seq data from Saunders et al., 2018. **b.** Correlation between expected and predicted cell proportions of test mixed transcriptional profiles simulated from scRNA-seq data. **c.** Heatmap of PCCs between cell type proportions estimated by each method in hippocampus spatial transcriptomics data. Abbreviations: CA: cornu ammonis.

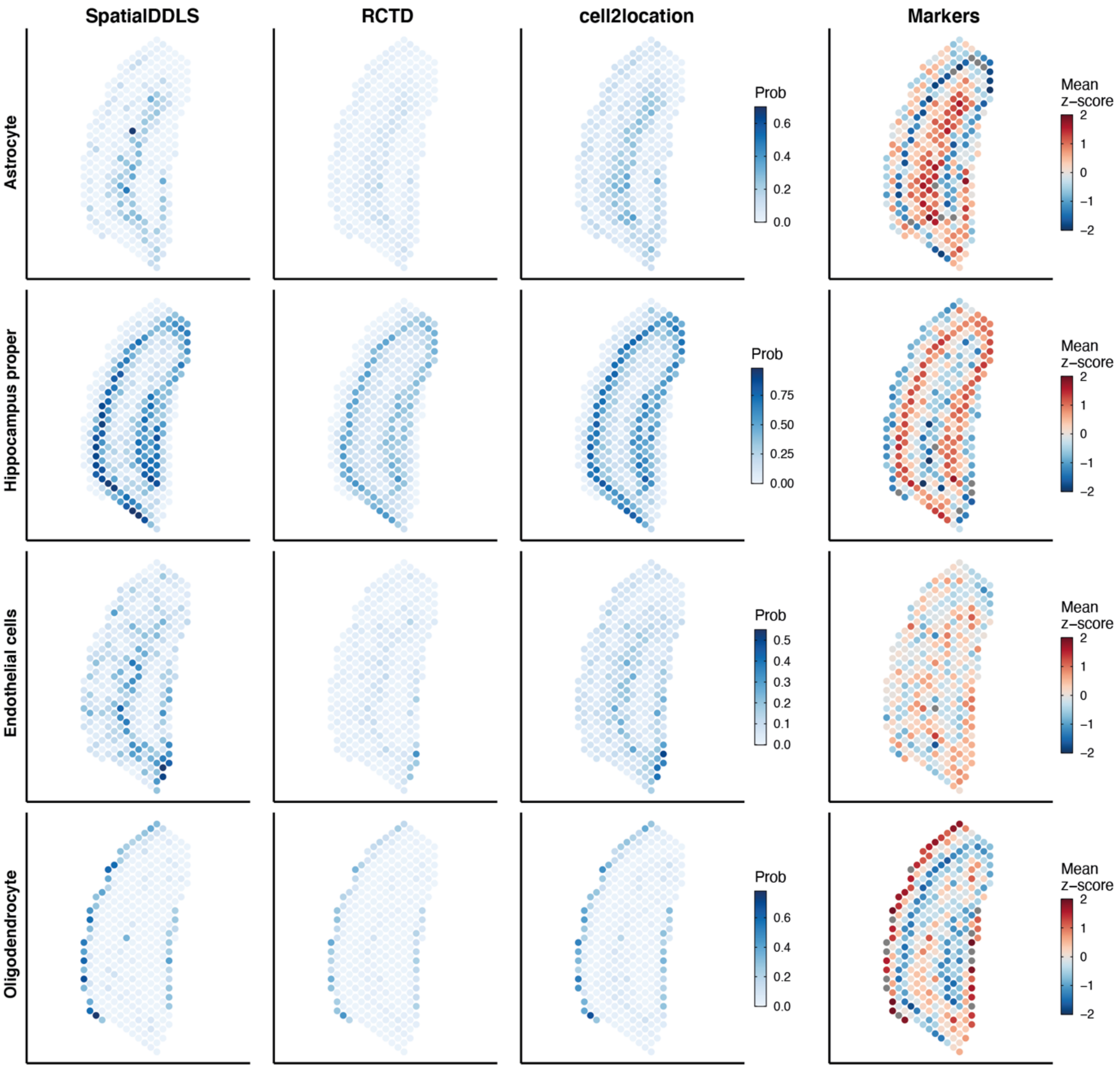

Figure S2: Estimated cell type proportions mapped to the spatial coordinates in the mouse hippocampus dataset. Endothelial cells and hippocampus proper were generated by adding up predicted proportions of: Endothelial cells = Endothelial stalk + Endothelial tip; Hippocampus proper = CA1 + CA3 + Dendate.

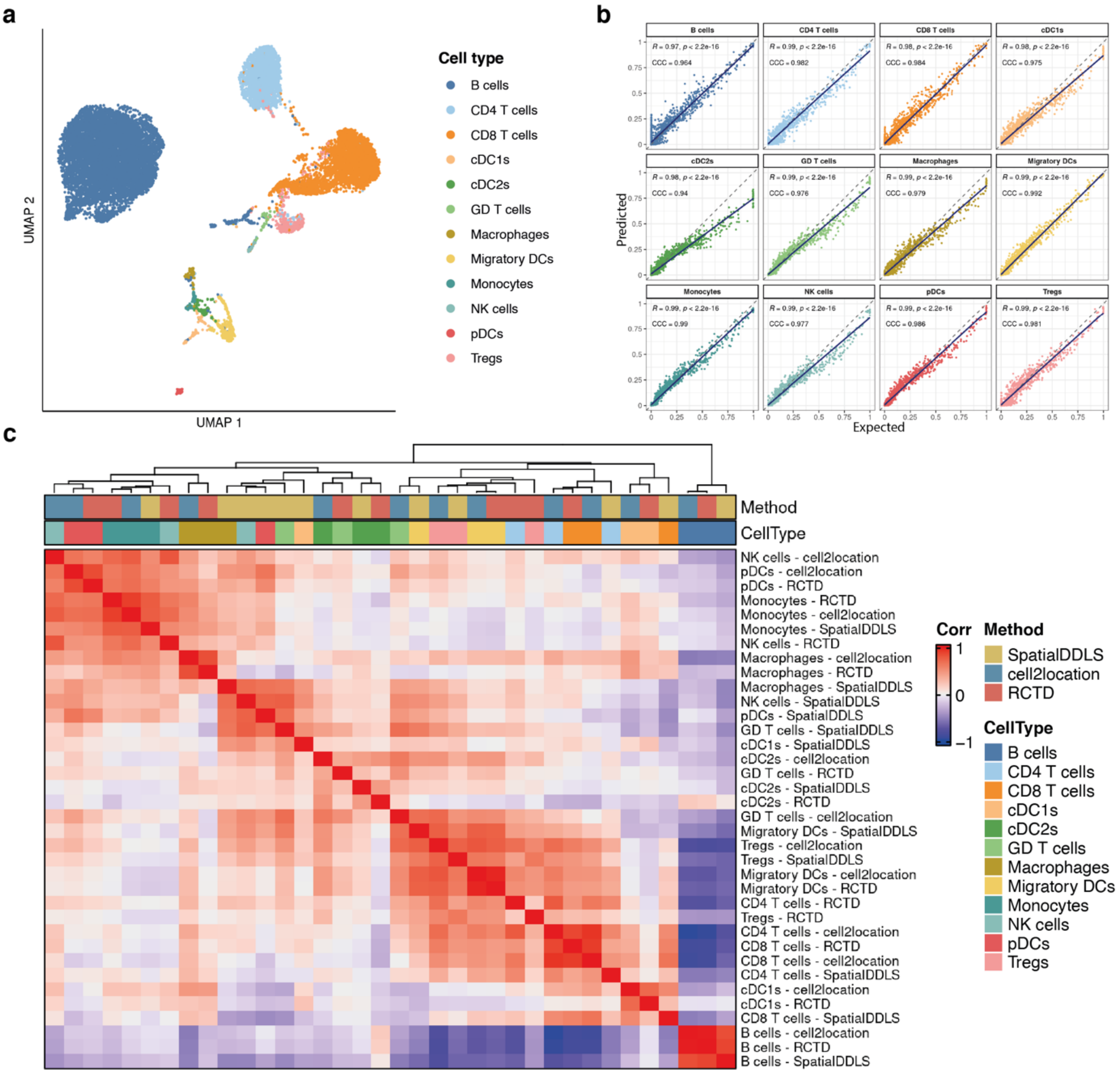

Figure S3: Mouse lymph node dataset. a. UMAP representation of scRNA-seq data from Lopez et al., 2022. **b.** Correlation between expected and predicted cell proportions of test mixed transcriptional profiles simulated from scRNA-seq data. **c.** Heatmap of PCCs between cell type proportions estimated by each method in lymph node spatial transcriptomics data. Abbreviations: cDC: conventional dendritic cells; GD: gamma-delta; DCs: dendritic cells; pDCs: plasmacytoid dendritic cells; Tregs: T regulatory cells.

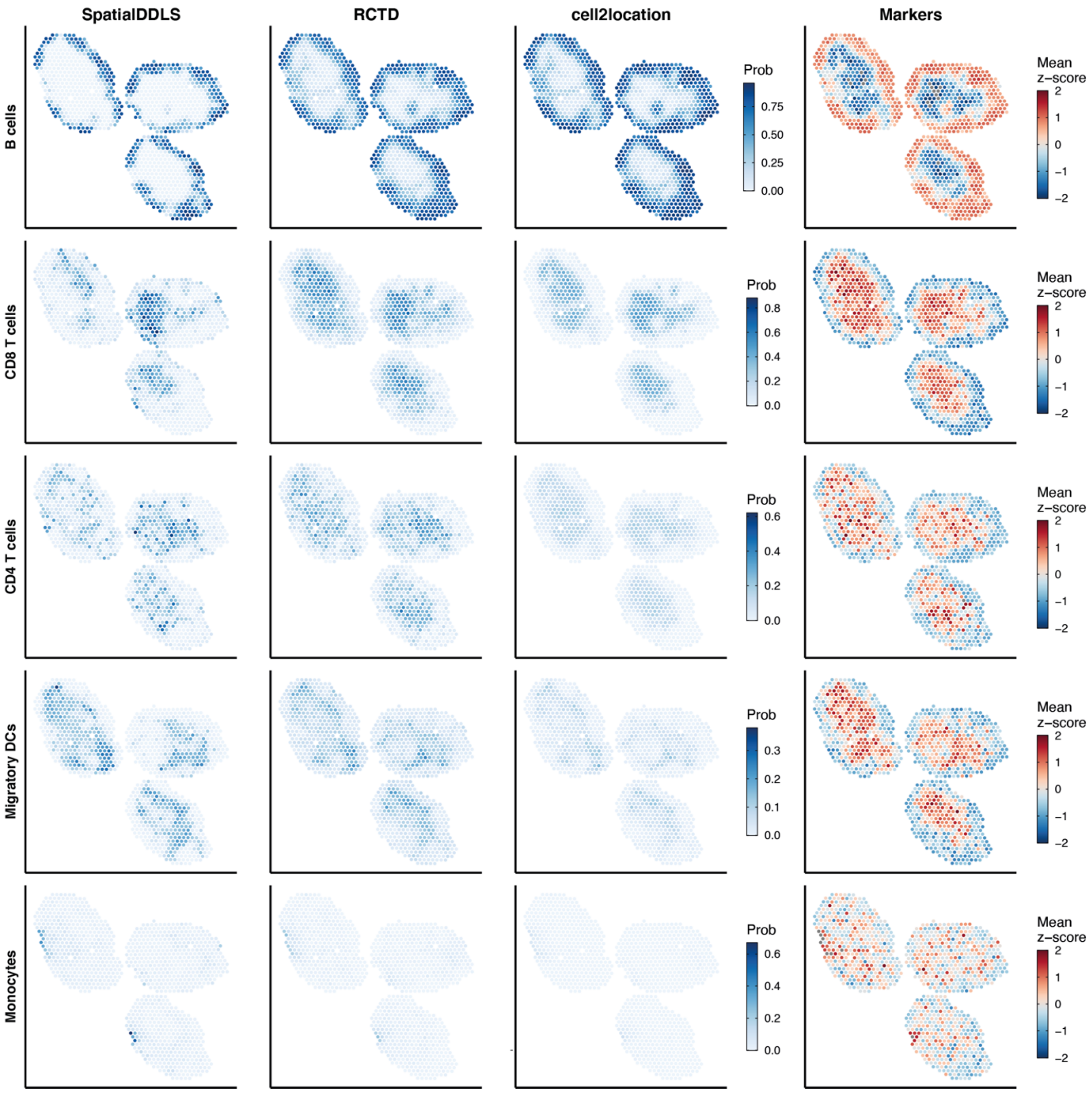

Figure S4: Estimated cell type proportions of representative cell types mapped to the spaptial coordinates in the mouse lymph node dataset. Abbreviations: DCs: dendritic cells.

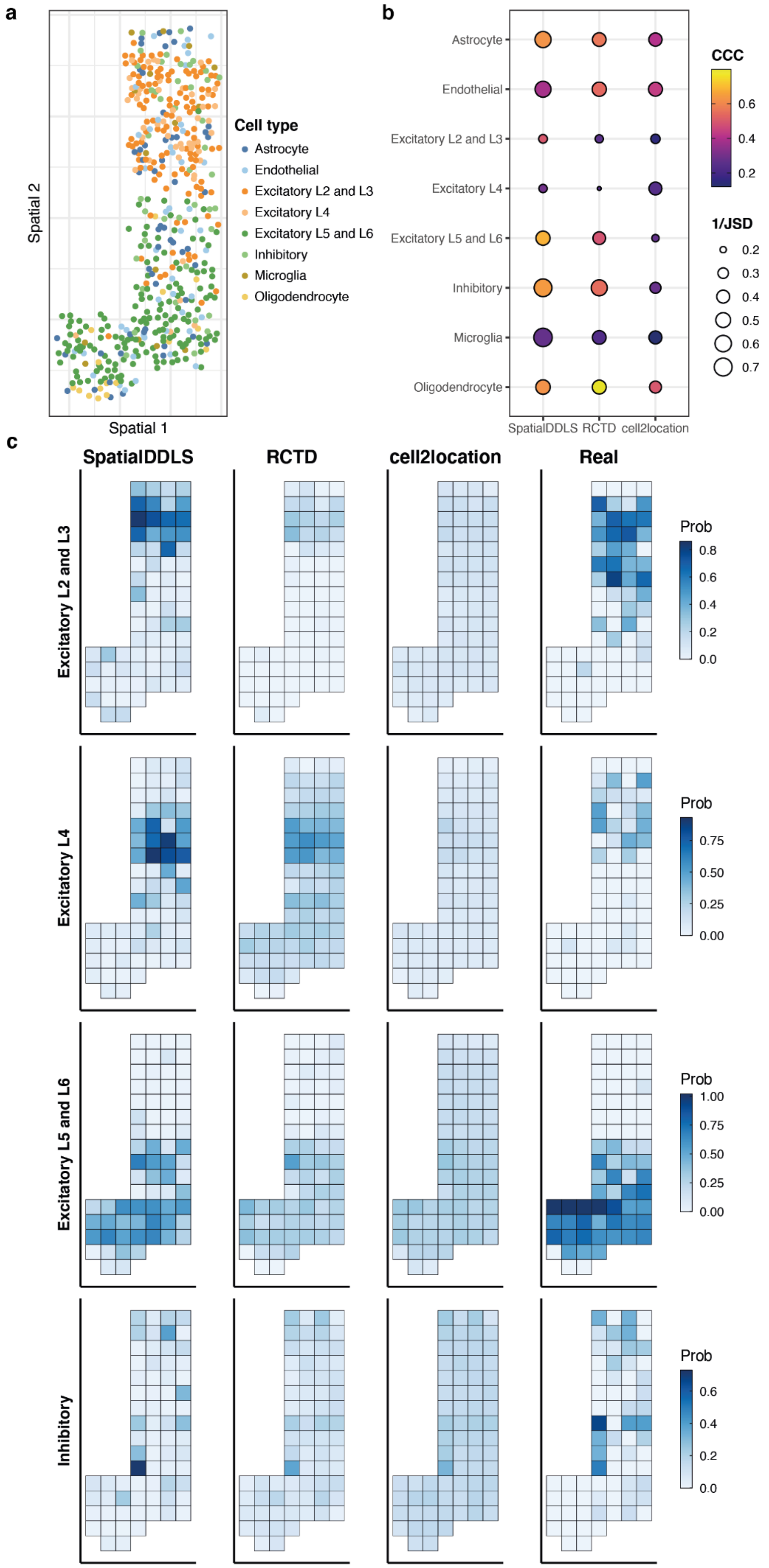

Figure S5: seqFISH of mouse brain cortex. **a.** seqFISH slide at the single-cell resolution annotated by cell type. **b.** Dotplot representing CCC (color) and 1/JSD (size) of each method for each cell type with respect to ground truth. **c.** Proportion of main cell types predicted by each method and ground truth in each simulated spot. Proportions within each row (sample cell type) are in the same color scale.

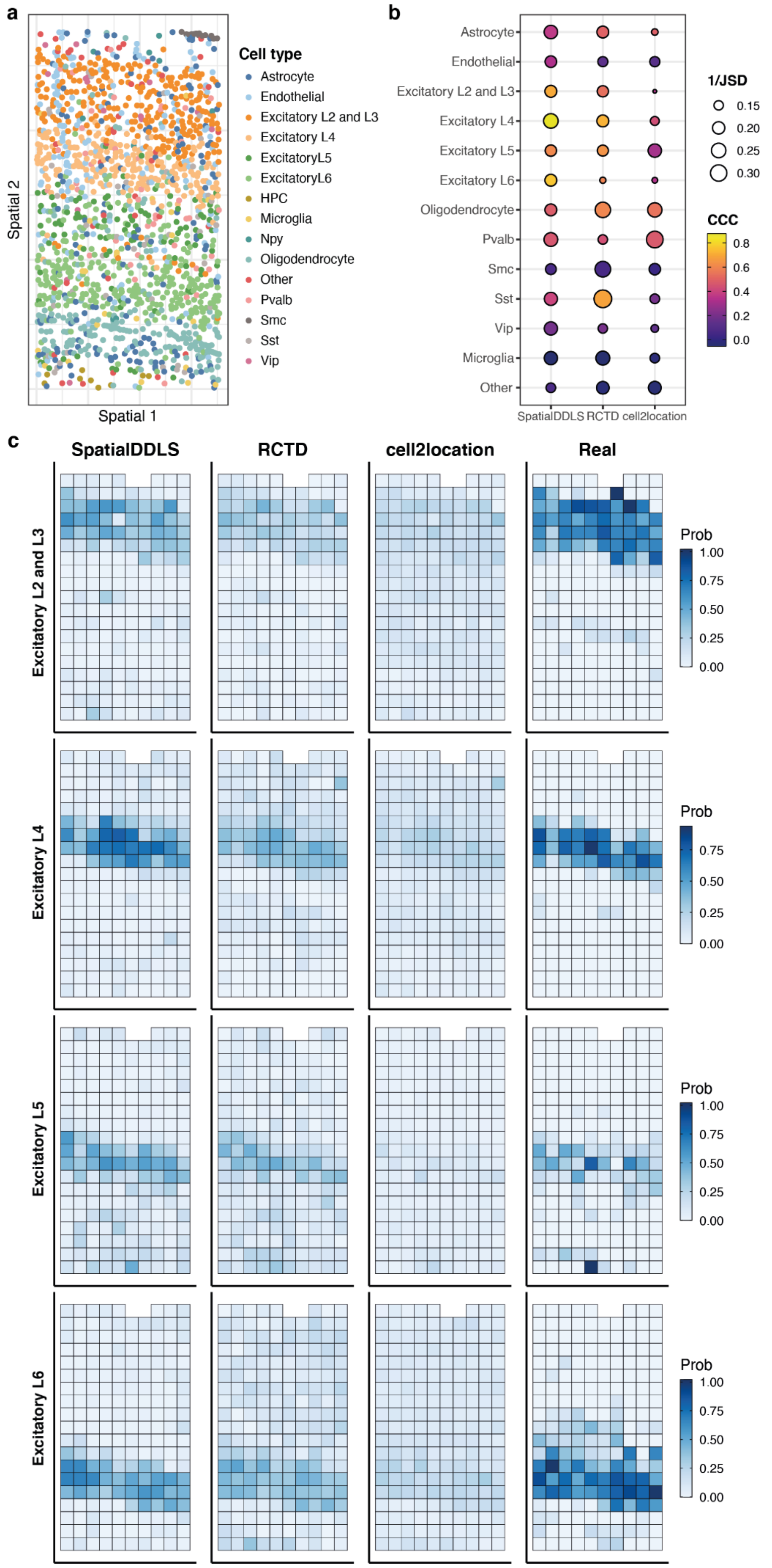

Figure S6: STARmap of mouse visual cortex. a. STARmap slide at the single-cell resolution annotated by cell type. **b.** Dotplot representing CCC (color) and 1/JSD (size) of each method for each cell type with respect to ground truth. **c.** Proportion of main cell types predicted by each method and ground truth in each simulated spot. Proportions within each row (sample cell type) are in the same color scale.

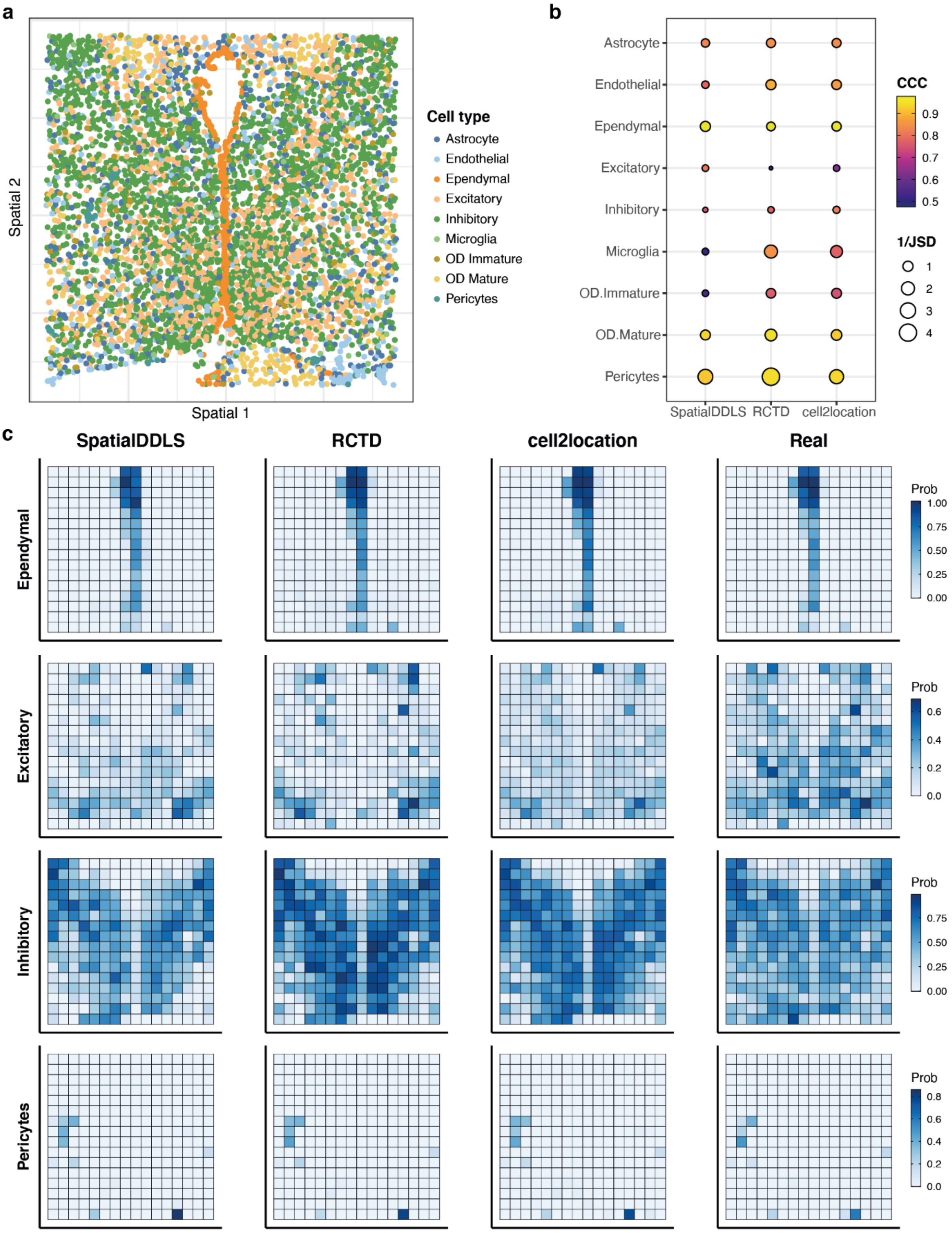

Figure S7: MERFISH of mouse MPOA. a. MERFISH slide at the single-cell resolution annotated by cell type. **b.** Dotplot representing CCC (color) and 1/JSD (size) of each method for each cell type with respect to ground truth. **c.** Proportion of main cell types predicted by each method and ground truth in each simulated spot. Proportions within each row (sample cell type) are in the same color scale.

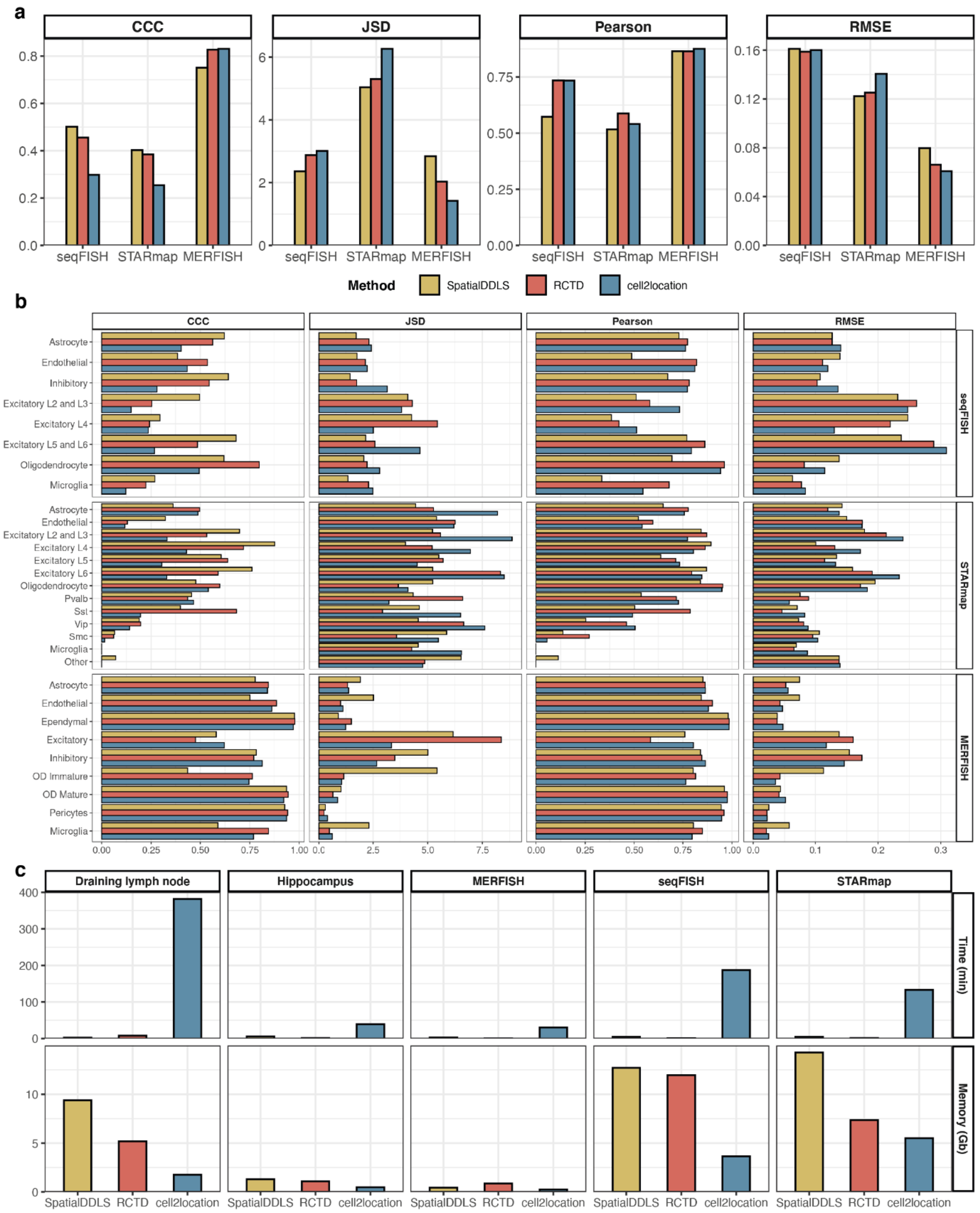

Figure S8: Benchmark results in single-resolution datasets. a. Mean results of each method for each ST dataset. **b.** Results of each method for each cell type and ST dataset**. c.** Running time and RAM consumption per method and ST dataset.

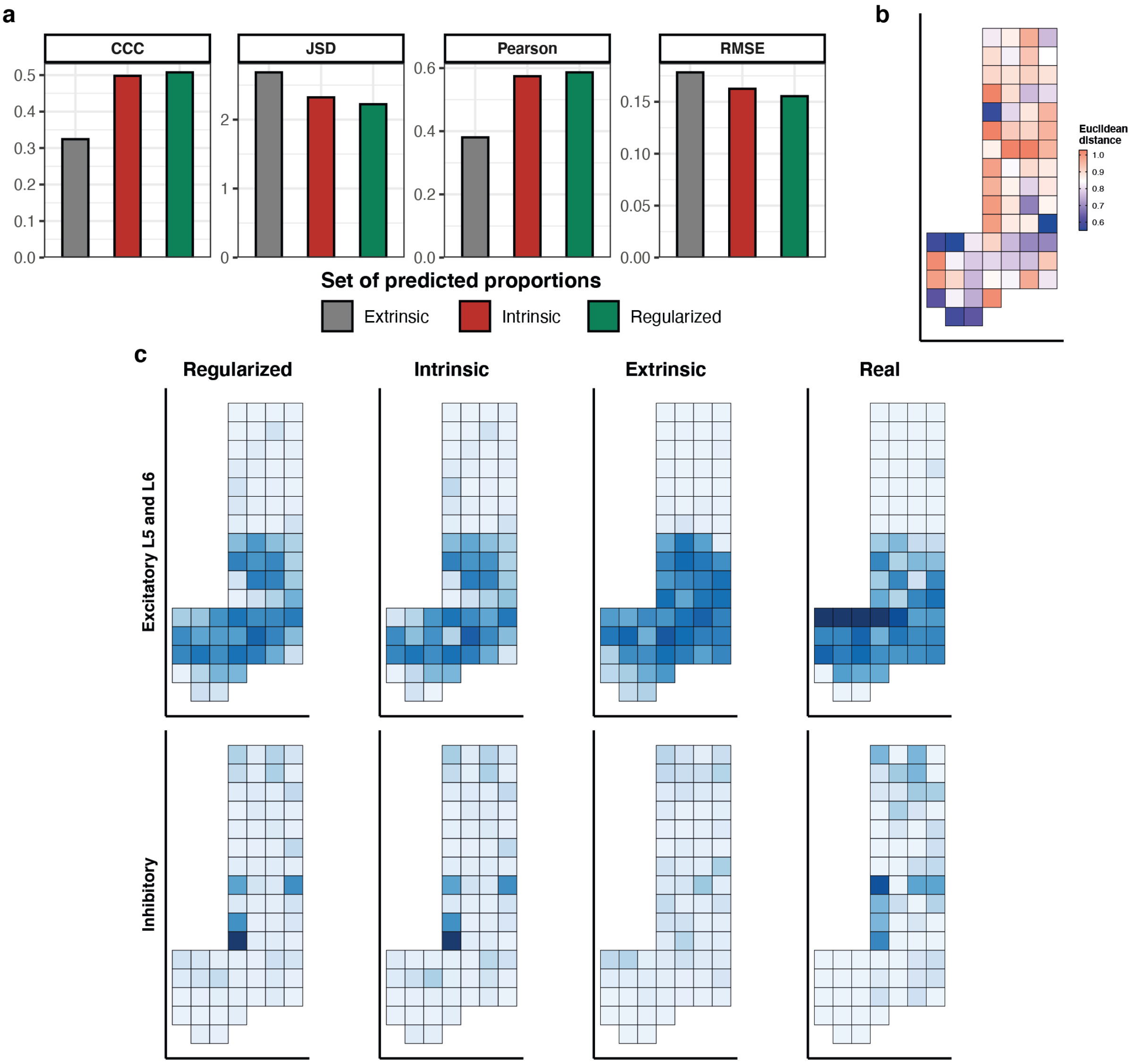

Figure S9: Spatial regularization. a. Performance metrics comparing each set of proportions with ground truth in the seqFISH+ ST dataset. **b.** Euclidean distances between intrinsic and extrinsic transcriptional profiles. **c.** Each set of proportions of Inhibitory and L5 and L6 neurons.

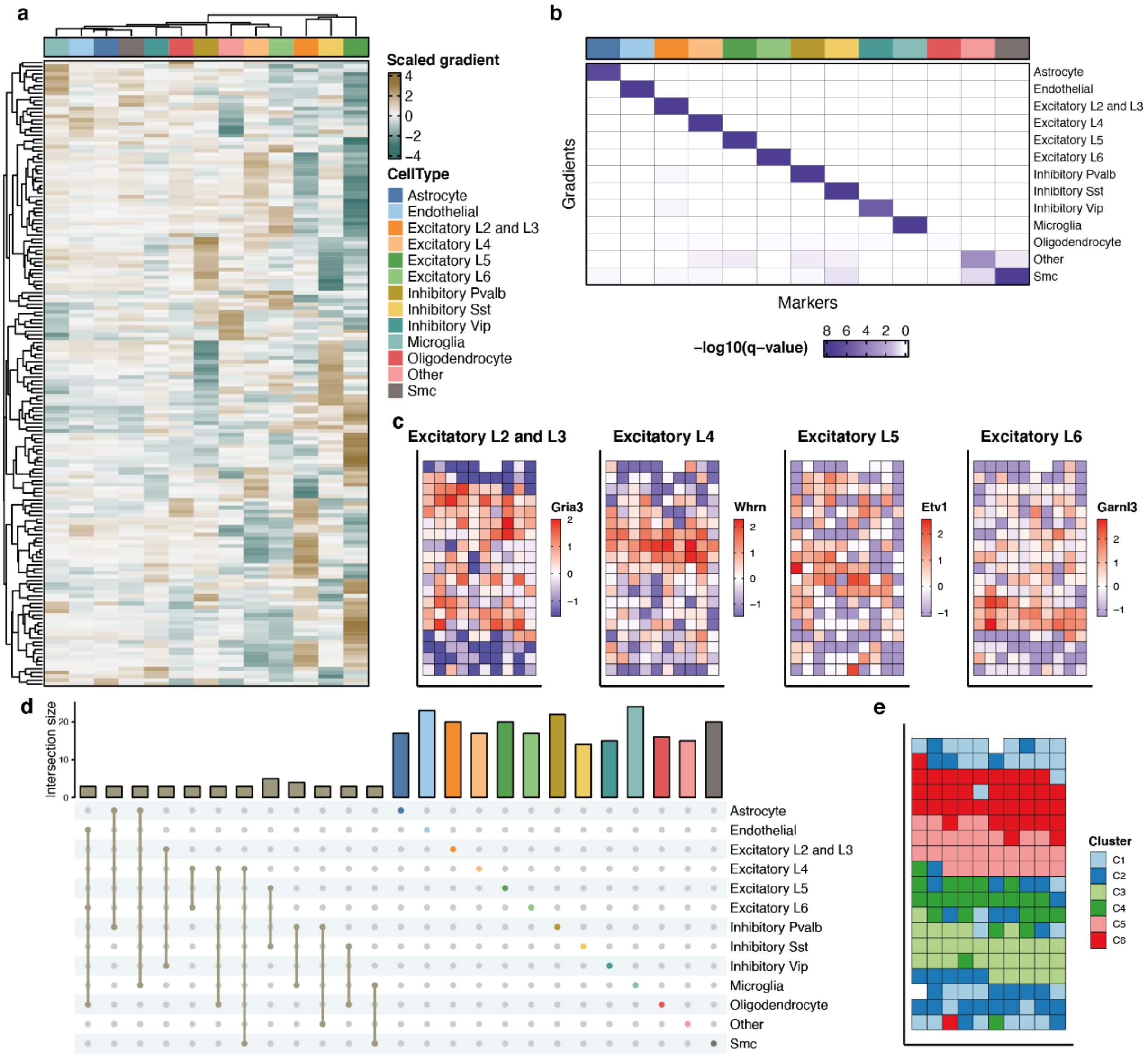

Figure S10: Additional modules in SpatialDDLS using STARmap of mouse visual cortex. a. Heatmap of the top 50 mean scaled gradient-based gene scores per cell type. **b.** Heatmap showing -log10(q-value) from overrepresentation analysis between gradient-based gene scores and markers per cell type. **c.** Scaled expression of 3 genes selected from the top gradient-based gene scores for Excitatory L2/L3, L4, L5 and L6 neurons. **d.** Upset plot of the top 50 genes according to gradient-based gene scores for each cell type. **e.** Clustering analysis based on predicted cell proportions.

Dataset	Cell type	Markers
Hippocampus	Astrocyes	Slc1a2 Apoe Aldoc
Hippocampus	Hippocampus proper	Nrgn Ywhaz
Hippocampus	Endothelial cells	Sparcl1 Ptgds Gpm6a
Hippocampus	Oligodendrocytes	Mbp Cnp Ptgds
Lymph node	B cells	Cd74 Cd19 Cd79a Cd79b Ly6d
Lymph node	CD4+ T cells	Cd4 Lef1 Fyb
Lymph node	CD8+ T cells	Cd8b1 Cd8a Trac
Lymph node	Migratory DCs	Ccl5 Anxa3 Fscn1

Table S1: Cell type markers used to check spatial location of main cell types.

References Supplementary Material

10x-Genomics, 2020. Visium spatial gene expression.

Eng, C.-H.L., Lawson, M., Zhu, Q., Dries, R., Koulena, N., Takei, Y., Yun, J., Cronin, C., Karp, C., Yuan, G.-C., Cai, L., 2019. Transcriptome-scale super-resolved imaging in tissues by RNA seqFISH+. Nature 568, 235–239. https://doi.org/10.1038/s41586-019-1049-y

Gu, Z., Eils, R., Schlesner, M., 2016. Complex heatmaps reveal patterns and correlations in multidimensional genomic data. Bioinformatics 32, 2847–2849. https://doi.org/10.1093/bioinformatics/btw313

Hao, Y., Hao, S., Andersen-Nissen, E., Mauck, W.M., Zheng, S., Butler, A., Lee, M.J., Wilk, A.J., Darby, C., Zager, M., Hoffman, P., Stoeckius, M., Papalexi, E., Mimitou, E.P., Jain, J., Srivastava, A., Stuart, T., Fleming, L.M., Yeung, B., Rogers, A.J., McElrath, J.M., Blish, C.A., Gottardo, R., Smibert, P., Satija, R., 2021. Integrated analysis of multimodal single-cell data. Cell 184, 3573-3587.e29. https://doi.org/10.1016/j.cell.2021.04.048

Li, B., Zhang, W., Guo, C., Xu, H., Li, L., Fang, M., Hu, Y., Zhang, X., Yao, X., Tang, M., Liu, K., Zhao, X., Lin, J., Cheng, L., Chen, F., Xue, T., Qu, K., 2022. Benchmarking spatial and single-cell transcriptomics integration methods for transcript distribution prediction and cell type deconvolution. Nat Methods 19, 662–670. https://doi.org/10.1038/s41592-022-01480-9

Li, H., Zhou, J., Li, Z., Chen, S., Liao, X., Zhang, B., Zhang, R., Wang, Y., Sun, S., Gao, X., 2023. A comprehensive benchmarking with practical guidelines for cellular deconvolution of spatial transcriptomics. Nat Commun 14, 1548. https://doi.org/10.1038/s41467-023-37168-7

Lopez, R., Li, B., Keren-Shaul, H., Boyeau, P., Kedmi, M., Pilzer, D., Jelinski, A., Yofe, I., David, E., Wagner, A., Ergen, C., Addadi, Y., Golani, O., Ronchese, F., Jordan, M.I., Amit, I., Yosef, N., 2022. DestVI identifies continuums of cell types in spatial transcriptomics data. Nat Biotechnol 40, 1360–1369. https://doi.org/10.1038/s41587-022-01272-8

Miller, B.F., Huang, F., Atta, L., Sahoo, A., Fan, J., 2022. Reference-free cell type deconvolution of multi-cellular pixel-resolution spatially resolved transcriptomics data. Nat Commun 13, 2339. https://doi.org/10.1038/s41467-022-30033-z

Moffitt, J.R., Bambah-Mukku, D., Eichhorn, S.W., Vaughn, E., Shekhar, K., Perez, J.D., Rubinstein, N.D., Hao, J., Regev, A., Dulac, C., Zhuang, X., 2018. Molecular, spatial, and functional single-cell profiling of the hypothalamic preoptic region. Science 362, eaau5324. https://doi.org/10.1126/science.aau5324

Pagès, H., 2021a. DelayedArray: A unified framework for working transparently with on-disk and in-memory array-like datasets. R package available at CRAN.

Pagès, H., 2021b. HDF5Array: HDF5 backend for DelayedArray objects. R package available at CRAN.

Risso, D., Perraudeau, F., Gribkova, S., Dudoit, S., Vert, J.-P., 2018. A general and flexible method for signal extraction from single-cell RNA-seq data. Nat Commun 9, 284. https://doi.org/10.1038/s41467-017-02554-5

Saunders, A., Macosko, E.Z., Wysoker, A., Goldman, M., Krienen, F.M., Rivera, H. de, Bien, E., Baum, M., Bortolin, L., Wang, S., Goeva, A., Nemesh, J., Kamitaki, N., Brumbaugh, S., Kulp, D., McCarroll, S.A., 2018. Molecular Diversity and Specializations among the Cells of the Adult Mouse Brain. Cell 174, 1015-1030.e16. https://doi.org/10.1016/j.cell.2018.07.028

Simonyan, K., Vedaldi, A., Zisserman, A., 2014. Deep Inside Convolutional Networks: Visualising Image Classification Models and Saliency Maps. https://doi.org/10.48550/arXiv.1312.6034

Wang, X., Allen, W.E., Wright, M.A., Sylwestrak, E.L., Samusik, N., Vesuna, S., Evans, K., Liu, C., Ramakrishnan, C., Liu, J., Nolan, G.P., Bava, F.-A., Deisseroth, K., 2018. Three-dimensional intact-tissue sequencing of single-cell transcriptional states. Science 361, eaat5691. https://doi.org/10.1126/science.aat5691

Yan, L., Sun, X., 2023. Benchmarking and integration of methods for deconvoluting spatial transcriptomic data. Bioinformatics 39, btac805. https://doi.org/10.1093/bioinformatics/btac805

Yu, G., Wang, L.-G., Han, Y., He, Q.-Y., 2012. clusterProfiler: an R Package for Comparing Biological Themes Among Gene Clusters. OMICS: A Journal of Integrative Biology 16, 284–287. https://doi.org/10.1089/omi.2011.0118
